# The political epidemiology of HIV

**DOI:** 10.7448/IAS.17.1.19327

**Published:** 2014-07-23

**Authors:** Joseph J Amon

**Affiliations:** Health and Human Rights Division, Human Rights Watch, New York, NY, USA

Since the start of the HIV pandemic, epidemiology has provided critical insights into the natural history of HIV infection, HIV prevalence and trends, and individual risk factors associated with infection. Epidemiology has also contributed to the evaluation of antiretroviral medicines and the development and assessment of public health interventions.

Less often, epidemiology has focussed upon understanding the influence of macro-social and economic factors that affect HIV prevalence, such as poverty, gender inequality, population mobility and conflict. Social epidemiology, examining the social and structural determinants of HIV vulnerability, has unquestionably contributed to advancing HIV understanding [1,2], yet dissatisfaction with what is construed as an overly biomedical approach to the HIV epidemic (and epidemiology) has led some to emphasize the need to go “beyond epidemiology” [3] and adopt ethnographic, “people-centered” approaches [4]. Epidemiologists too have periodically lamented the direction of the discipline and called for a more “consequentialist” approach [5,6].

To be consequential, in terms of understanding the HIV epidemic and developing effective responses, epidemiologic research must recognize the significant influence of politics and political determinants *– laws, policies and their enforcement –* on health-related behaviours and outcomes. There are many examples of the important role of politics and political leadership in the response to HIV, including the notoriously negative (e.g. HIV denialism in South Africa, the promotion of fake “cures” for HIV in the Gambia and disastrous “wars” on people who use drugs in many countries [7–9]) and the positive (e.g. the response to HIV in Brazil, which embraced civil society activism, universal access to care and the adoption of health policies grounded in respect for human rights and recognizing the need to combat stigma and discrimination [10]). However, the engagement of epidemiology in examining the consequences of these factors, with some exceptions [11], has been underwhelming.

Similarly, despite recognition of the role of the law and law enforcement in HIV vulnerability and access to treatment, especially where key populations such as people who use drugs, men who have sex with men (MSM), and sex workers are criminalized [12], epidemiology (including social epidemiology) has paid, at best, uneven attention to the broad influence of government health, education, drug, criminal justice and other laws, policies and enforcement practices on health [13–18]. Political factors are sometimes obscured in multi-level models that blur the responsibility of governments (for abusive laws) with complex “social” factors less clearly tied to specific actors. By contrast, the development of an explicit *political epidemiology*, distinct from social epidemiology, would highlight, for example, how high levels of police harassment and abuse, discriminatory laws and practices, and policies that deny prevention information and services impact vulnerability to HIV infection, access to treatment and AIDS mortality. Epidemiologic investigations of political determinants would also strengthen our understanding of causal relationships between human rights abuses and health outcomes.

In two decades of work on HIV and human rights, Human Rights Watch has documented the experiences of criminalized populations, adolescents, women and people living with HIV and AIDS as they confront discrimination in healthcare settings, employment and housing, and violence in their own homes and communities. Our research has examined restrictions on free speech and on the ability of civil society to register as nongovernmental organizations seeking to provide HIV information and services. We have documented the consequences of rape, domestic violence and homophobic attacks; and arbitrary arrests, beatings and torture of people who use drugs, gay and bisexual men, sex workers and other vulnerable groups in detention settings. Our analysis has shown the ways in which these injustices – based in laws and policies, and law enforcement, or the failure to enforce laws that could protect vulnerable populations – facilitate HIV transmission and reduce access to life-saving treatment.

We have used both quantitative and qualitative methods to examine the impact of law enforcement on HIV vulnerability, finding for example that 36% of individuals in New Orleans we surveyed who exchanged sex for money, drugs or life necessities carried fewer condoms than they needed for fear of trouble from the police [19]. In New York, we interviewed a sex worker who explained how police harassment limited the effectiveness of the city's free condom distribution programme: “If I took a lot of condoms, the police would arrest me. If I took a few or only one, I would run out and not be able to protect myself. How many times have I had unprotected sex because I was afraid of carrying condoms? Many times” [20].

In Zambian prisons, where condoms are not permitted, high rates of HIV and sexual violence might best be addressed by criminal justice reforms to reduce the incarceration of non-violent prisoners and individuals awaiting trial. Yet we found that 95% of juveniles, 88% of adult males and 75% of adult females we interviewed had been continuously detained from the time of their arrest, without access to police bond or bail. Two inmates reported having been held on remand for six years, and one reported having been held for 10 years before conviction [21].

In Jamaica, we examined the impact of anti-sodomy laws, which, though rarely enforced, contribute to a climate of fear and stigmatization of MSM. We found that these laws had been used by public television stations to justify their refusal to air public service announcements addressing homophobia, and by landlords to justify their refusal to rent apartments to lesbian, gay, bisexual and transgendered (LGBT) persons. Many of the young MSM and transgender people we interviewed had been expelled from their homes as teenagers; some ended up on the streets and engaged in sex work [22]. In Thailand, a drug user living with HIV said that he was told that his drug use rendered him ineligible for life-saving HIV treatment: “The doctor said if I use drugs, I can't have ART” [23].

Our research in countries such as China [24] and Russia [25] found that laws and government policies deny key populations evidence-based HIV prevention information. In Senegal, police harassed nongovernmental organizations providing outreach services [26]. In Uganda [27] and the United States [28], adolescents were denied access to comprehensive sex education and HIV prevention information in schools.

Most definitions of epidemiology describe two core functions: examining the distribution and determinants of health, and acting on this knowledge to promote health. Similarly, political epidemiology seeks to understand political determinants of health, and through their link to the human rights obligations of governments – to refrain from interfering with access to prevention or treatment, protect individuals from harm, expand access to care and fulfil the right to health through evidence-based HIV programmes targeting those most affected [29] – to encourage action to promote health. Political epidemiology research can also support the development of novel public health interventions [30,31], including the evaluation of programmes providing legal services (e.g. addressing intimate-partner violence or discrimination), legal literacy campaigns (“know your rights”) and training of police and healthcare providers (e.g. on the right to access HIV prevention, confidentiality and consent in HIV testing, and post-rape care and post-exposure prophylaxis) [32].

Of course, political determinants are not the only factors influencing HIV vulnerability. However, in the context of limited resources, political epidemiological research can help identify targeted, cost-effective, HIV intervention strategies, and investing in research on the political epidemiology of HIV can help ensure that behavioural and biomedical HIV interventions reach their targeted populations, and that individuals are protected not only from HIV but from human rights abuses more broadly.
